# Optimization of the Ultrasonic-Assisted Extraction of Bioactive Flavonoids from *Ampelopsis grossedentata* and Subsequent Separation and Purification of Two Flavonoid Aglycones by High-Speed Counter-Current Chromatography

**DOI:** 10.3390/molecules21081096

**Published:** 2016-08-20

**Authors:** Hongbing Zhang, Guoyong Xie, Mei Tian, Qian Pu, Minjian Qin

**Affiliations:** Department of Resources Science of Traditional Chinese Medicines, State Key Laboratory of Modern Chinese Medicines, China Pharmaceutical University, Nanjing 210009, China; flood198688@163.com (H.Z.); ivytian11@hotmail.com (M.T.); 15651721977@163.com (Q.P.)

**Keywords:** *Ampelopsis grossedentata*, ultrasonic-assisted extraction, response surface methodology, flavonoids, high-speed counter-current chromatography

## Abstract

The fermented leaf of *Ampelopsis grossedentata* has been used as a beverage and folk medicine called “vine tea” in the southern region of China. In this paper, the optimum extraction conditions for the maximum recovery amounts of total flavonoids (TF), dihydromyricetin (DMY), myricitrin (MYG) and myricetin (MY) from natural *Ampelopsis grossedentata* leaves subjected to ultrasonic-assisted extraction (UAE) were determined and optimized by using response surface methodology. The method was employed by the Box–Behnken design (BBD) and Derringer’s desirability function using methanol concentration, extraction time, liquid/solid ratio as factors and the contents of TF, DMY, MYG and MY as responses. The obtained optimum UAE conditions were as follows: a solvent of 80.87% methanol, an extraction time of 31.98 min and a liquid/solid ratio of 41.64:1 mL/g. Through analysis of the response surface, it implied that methanol concentration and the liquid/solid ratio had significant effects on TF, DMY, MYG and MY yields, whereas extraction time had relatively little effects. The established extraction and analytical methods were successfully applied to determine the contents of the total flavonoids and three individual flavonoids in 10 batches of the leaf samples of *A. grossedentata* from three counties in Fujian Province, China. The results suggested the variability in the quality of *A. grossedentata* leaves from different origins. In addition, high purities of dihydromyricetin and myricetin were simultaneously separated and purified from the extract subjected to optimized UAE, by high-speed counter-current chromatography using a solvent system of *N*-hexane–ethyl acetate–methanol–water (1:3:2:4; *v*/*v*/*v*/*v*). In a single operation, 200 mg of the extract were separated to yield 86.46 mg of dihydromyricetin and 3.61 mg of myricetin with the purity of 95.03% and 99.21%, respectively. The results would be beneficial for further exploiting the herbal products and controlling the quality of the herb and its derived products.

## 1. Introduction

**Figure 1:**
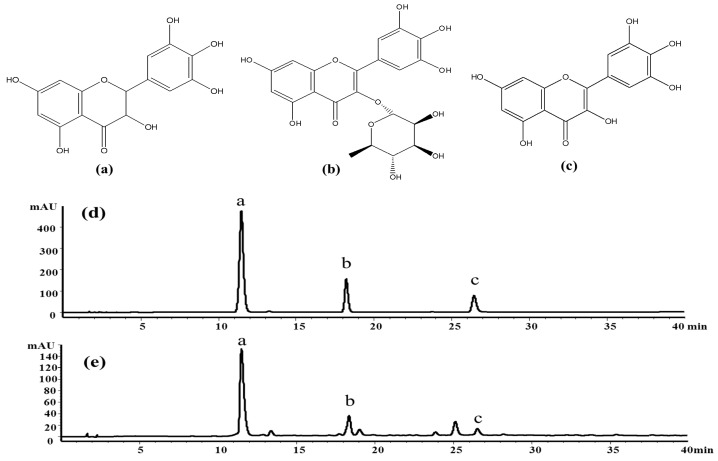
Chemical structures of dihydromyricetin (**a**), myricitrin (**b**), myricetin (**c**) and the typical chromatograms of the standard mixture solution (**d**); and a sample extract obtained by ultrasonic-assisted extraction (UAE) (**e**). The chromatograms were acquired by a high performance liquid chromatography instrument equipped with a diode-array detector at 292 nm.

*Ampelopsis grossedentata* (Hand.-Mazz.) W.T. Wang is a medicinal and edible plant growing in mountainous areas of southern China, and its fermented leaf has been used as a beverage and folk medicine called “vine tea”. It has been reported that vine tea contains a great amount of flavonoids [1,2], which possess a number of biological activities, such as hypoglycemic [3,4], antioxidant [5,6,7,8], anti-thrombosis function [9], anti-tumor [10,11], anti-inflammatory [12] and antibacterial activities [13]. Among the flavonoids, dihydromyricetin (a), myricitrin (b) and myricetin (c) have been confirmed as the main bioactive constituents of the herb (Figure 1). According to recent pharmacological studies, dihydromyricetin and myricetin exhibit broad-spectrum antibacterial activities, anti-inflammatory effects [3,14] and antioxidant capacities [8,15] and are used to cure diabetes [16], cancer and hypertension [10,17,18]. However, it is difficult to purify dihydromyricetin and myricetin from the extract of *A. grossedentata* leaves, although the extract contains a high percentage of the two compounds [19].

Extraction is a very important process for herb products’ development and their quality control. A variety of extraction methods have been utilized for the separation and analysis of flavonoids from *A. grossedentata*. Li et al. reported a microwave multi-stage countercurrent extraction method for extracting dihydromyricetin from *A.* *grossedentata* leaves [20]. However, the quality evaluation marker for the extraction efficiencies of that study was only one compound, which could not reflect the whole quality of the extract. Ying et al. using the supercritical carbon dioxide method extracted flavonoids and phenolics from *A. grossedentata* leaves [6]. However, the extraction efficiencies of those methods are not satisfactory. The lower yields and lack of appropriate analytic methods remain issues to be solved.

Ultrasonic-assisted extraction (UAE) is a simple, rapid and effective extraction technique that uses ultrasonic waves to generate a cavitation in the solvent, which accelerates a much higher penetration of solvent into the raw plant materials [21]. Compared to other conventional extraction techniques, UAE is a greener methodology that allows for a high reproducibility in a shorter time, simplified manipulation, significant reduction in organic solvent consumption and temperature, lower energy input and higher efficiency [22]. When a new UAE method is developed, optimization of the extraction conditions, such as solvent type, extraction time and sample-solvent ratio, is indispensable for the best extraction effect within the shortest time [23].

Response surface methodology (RSM) is a collection of statistical and mathematical techniques for developing, improving and optimizing processes [24], which is a valuable tool to investigate the interaction between factors and quantitatively depict the effects of given parameters on their measured responses [23,25]. Box–Behnken design (BBD), a commonly-used method of RSM, is efficient and makes it easier to arrange and interpret the optimization experiments [26]. Thus, in the present study, BBD was employed to investigate the effects of various process variables, including methanol concentration, extraction time and liquid/solid ratio, on the yields of the total flavonoid (TF), dihydromyricetin (DMY), myricitrin (MYG) and myricetin (MY) from *A. grossedentata* subjected to UAE. The main objectives of the present study were to determine the critical variables, as well as the optimal extraction conditions that would allow maximum response yields in accordance with the response surface and contour plots.

High-speed counter-current chromatography (HSCCC) is a support-free liquid-liquid partition chromatographic technique that uses as a stationary phase a liquid matrix instead of the common-used solid support matrix, preventing the reversible adsorption of the samples onto the solid supports [27]. It offers various advantages, such as high solute loading capability, high recovery, high repeatability and low solvent consumption [28]. Therefore, HSCCC is a good alternative as a preparative technique to improve the separation of flavonoids, as the traditional separation methods, such as silica gel column chromatography, are often time consuming and can easily result in the adsorption of the sample onto the solid support, resulting in low yields [27,29]. Therefore, in this paper, a high-speed counter-current chromatography (HSCCC) method has been successfully established to simultaneously separate and purify dihydromyricetin and myricetin with high purity from the extracts of *A. grossedentata* leaves under the optimal UAE conditions.

## 2. Results and Discussion

### 2.1. Selection of the UAE Parameters’ Ranges by Single Factor Tests

The extraction yield of constituents from crude plant materials was affected by many factors. Thus, a selection of the appropriate extraction solvent or the extraction method is a key consideration. Based on the results of the preliminary experiments shown in Supplementary Materials (Table S1), methanol and UAE were selected as reasonable options for the extraction of flavonoids from *A. grossedentata* leaves.

When utilizing methanol and UAE as the extraction solvent and the extraction method, respectively, factors such as solvent concentration, extraction time and liquid/solid ratio are generally considered as having significant effects on the extraction efficiency. For this study, an initial step was performed to screen for the active factors influencing the experimental responses. The aqueous methanol concentration (20%–100%), extraction time (10–50 min) and the liquid/solid ratio (10:1–50:1 mL/g) were investigated using single factor tests to roughly select the influential ranges for in-depth investigations. The amounts of total flavonoids, dihydromyricetin, myricitrin and myricetin were investigated for each factor, while other factors were held at a constant value.

The appropriate aqueous methanol concentration range was the first factor investigated. The extraction yield for total flavonoids, dihydromyricetin, myricitrin and myricetin were compared under different concentrations of *v*/*v* (20%, 40%, 60%, 80% and 100%). The concentration of aqueous methanol (80% *v*/*v*) was determined to be the most effective for extraction efficiency. This is likely due to the changes in solvent polarity as the *v*/*v* concentration of methanol is altered. Specifically, the principle of similarity and intermiscibility suggests that a plant ingredient is easily dissolved when the polarities of the solvent and solute are similar. Therefore, an 80% *v*/*v* aqueous methanol concentration was reasonable for subsequent experiments.

The time range required for ultrasonic extraction was the second factor investigated. The extraction yields of the total flavonoids and three individual flavonoids were compared following ultrasonic extraction with 80% methanol for 10, 20, 30, 40 and 50 min. The results indicated that the maximum value for extraction efficiency was reached after 30 min and then leveled off. 

The most appropriate liquid/solid ratio range was the third factor to be investigated. Liquid/solid ratios of 10:1, 20:1, 30:1, 40:1 and 50:1 (mL/g) were utilized for contents of the total flavonoids and three individual flavonoids, while maintaining a methanol concentration of 80% (*v*/*v*) and an ultrasonic extraction time of 30 min. The extraction efficiency decreased when the liquid/solid ratio rose from 40:1–50:1 (mL/g).

Although UAE parameters’ ranges had been screened by single factor tests, but this conventional method ignores the combined interactions among various physical and chemical parameters, and the evaluation of possible interaction effects arising between factors is still difficult, while misleading inferences may occur. To further optimize UAE parameters, the BBD test was used for the subsequent RSM study.

Based on the above single factor tests, we adopted a methanol concentration range of 60%–100% (*v*/*v*), an extraction time range of 20–40 min and a liquid/solid ratio range of 30:1–50:1 mL/g for subsequent RSM experiments (Table 1). 

### 2.2. RSM Experimental Design and Model Fitting

A Box–Behnken design (BBD) is one of the RSM methods used to examine the relationship between one or more response variables and a set of quantitative experimental parameters [30]. The BBD method (three variables and three levels with 17 runs) was selected in the present study in order to optimize the extraction conditions for the maximum recovery of bioactive flavonoids from *A. grossedentata* leaves. According to the principle of the BBD, the three independent variables were methanol concentration (X_1_), extraction time (X_2_) and liquid/solid ratio (X_3_). In addition, the low, middle and high levels of each independent variable were screened by the single factor tests and were designated as coded terms −1, 0 and +1, respectively (Table 1). Regression analysis was performed according to the experimental data and was fitted to the second-order polynomial model to express the content of TF, DMY, MYG and MY as a function of the independent variables as follows:

(1) $$Y=b_{0}+\sum_{i=1}^{3}b_{i}X_{i}+\sum_{i=1}^{3}b_{ii}X_{i}^{2}+\sum_{i\neq j=1}^{3}b_{ij}X_{i}X_{j}$$

where *Y* is the measured response variable, *b*_0_ is a constant, *bi*, *b_ii_* and *b_ij_* are the linear, quadratic and interaction coefficients, respectively, and *X_i_* and *X_j_* are the levels of the independent variables.

The Design Expert software (Version 8.0.6, Stat-Ease Inc., Minneapolis, MN, USA) was used for the experimental design, regression analysis and analysis of variance (ANOVA) of the BBD test. Three-dimensional surface response plots were generated by changing two variables within the experimental range and holding the other variable constant at the central point. The fitness of the polynomial model equation to the responses was evaluated by the coefficient of *R* square, as well as by the lack of fit using the *F*-test.

**Figure 2:**
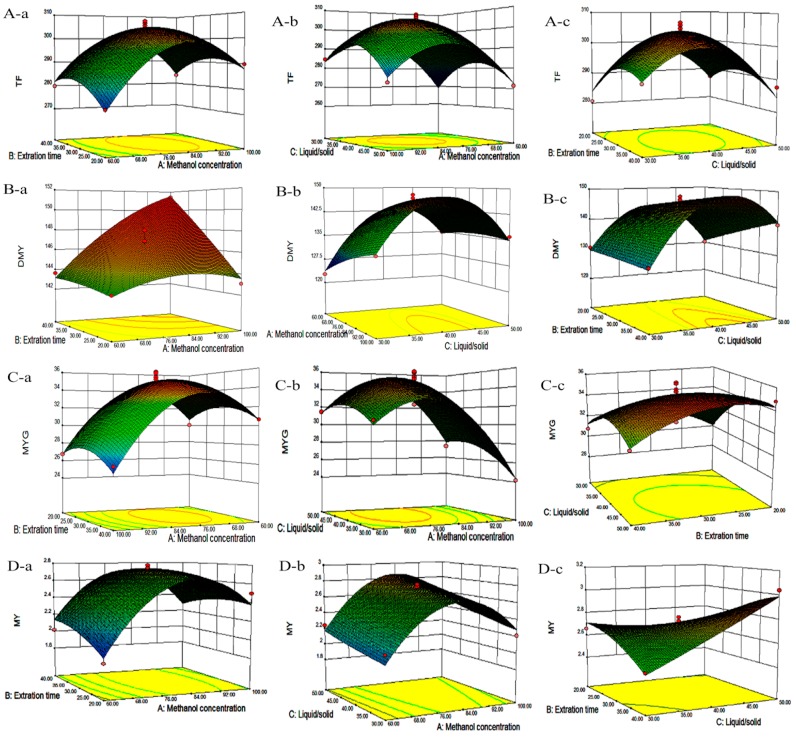
Response surface plots and contour plots show the effects of (**a**) methanol concentration and extraction time, (**b**) methanol concentration and liquid-solid and (**c**) extraction time and liquid-solid on the response of the amounts of total flavonoids (**A**); dihydromyricetin (**B**); myricitrin (**C**) and myricetin (**D**).

The second order polynomial model is the empirical model most commonly used for engineering processes and optimization methodology. The amounts of total flavonoids and three individual flavonoids measured in the extracts of *A. grossedentata* leaves from all 17 experimental runs of the BBD test were shown in Table 1. The quality of the generated model was evaluated by analysis of variance (ANOVA), *F*-value and the lack of fit of the model. The ANOVA results suggested that the generated models had very high *F*-values and very low *p*-values (0.0001) for all four responses (Table 2). In addition, the high coefficient of determination *R* square values and the values of insignificance of lack of fit (*p* > 0.05) were observed in Table 2, indicating that the quadratic models were highly significant to the obtained data and capable of describing the relationship between the extraction conditions and responses during the UAE process. The fitted quadratic models for yields of TF, DMY, MYG and MY are given in Equations (2)–(5), respectively. Three dimensional surface and contour plots were generated based on Equations (2)–(5) and are shown in Figure 2A–D, which presents the relationship between every two process variables in each response.

Y_TF_ = 304.31 + 4.36X_1_ +1.24X_2_ − 0.96X_3_ − 3.07X_1_X_2_ − 2.79X_1_X_3_ − 2.40X_2_X_3_ − 17.02X_1_^2^ − 5.54X_2_^2^ − 12.18X_3_^2^ (2)

Y_DMY_ = 146.50 + 2.35X_1_ + 2.16X_2_ + 3.25X_3_ + 1.83X_1_X_2_ − 2.13X_1_X_3_ + 2.70X_2_X_3_ − 1.12X_1_^2^ − 0.20X_2_^2^ − 13.90X_3_^2^ (3)

Y_MYG_ = 34.80 − 2.28X_1_ − 0.33X_2_ + 1.21X_3_ − 0.32X_1_X_2_ + 0.55X_1_X_3_ − 0.89X_2_X_3_ − 4.99X_1_^2^ − 1.45X_2_^2^ − 1.44X_3_^2^ (4)

Y_MY_ = 2.73 + 0.16X_1_ + 0.037X_2_ + 0.10X_3_ − 0.085X_1_X_2_ + 0.22X_1_X_3_ + 0.17X_2_X_3_ − 0.49X_1_^2^ − 0.062X_2_^2^ + 0.010X_3_^2^ (5)

### 2.3. Analysis of Response Surface

#### 2.3.1. Effects of Process Variables on Total Flavonoids Content 

**Figure 3:**
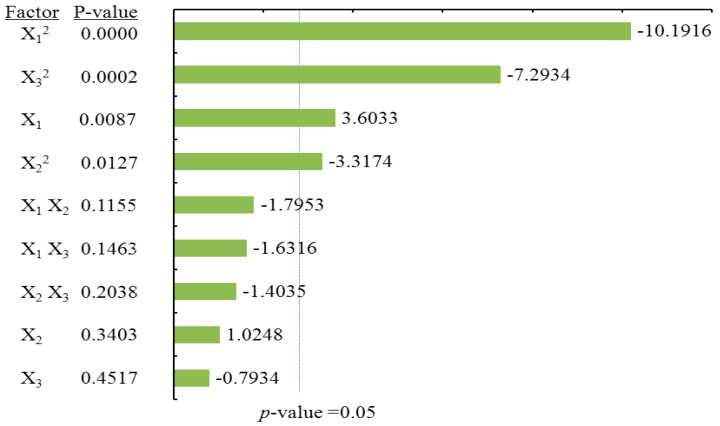
Pareto chart of the effects for the total flavonoids yield. Standardized effect estimate (absolute value).

**Figure 4:**
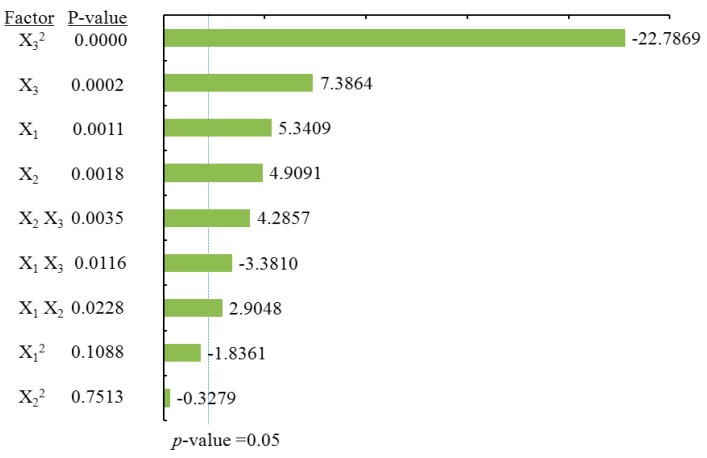
Pareto chart of the effects for the dihydromyricetin yield. Standardized effect estimate (absolute value).

**Figure 5:**
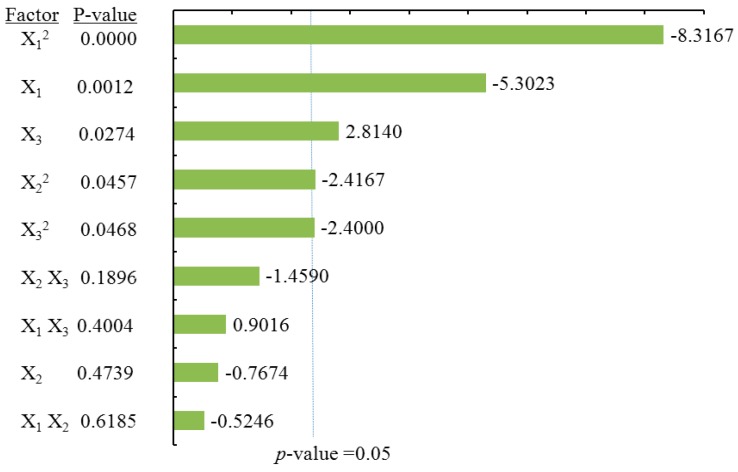
Pareto chart of the effects for the myricitrin yield. Standardized effect estimate (absolute value).

**Figure 6:**
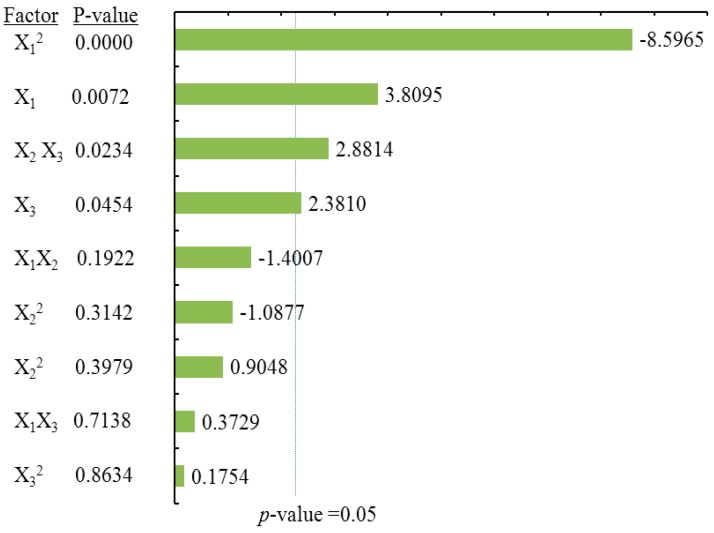
Pareto chart of the effects for the myricetin yield. Standardized effect estimate (absolute value).

To visualize the effects of process variables on the yields of total flavonoids and individual flavonoids, the Pareto charts were drawn (Figure 3, Figure 4, Figure 5 and Figure 6) by Minitab (Version 17.1). The Pareto chart illustrates the effects of the individual parameters and their interactions. The length of each bar is proportional to the absolute value of the associated regression coefficient or estimated effect. The effects of all parameters and interactions were standardized (each effect was divided by its standard error). The order in which the bars are displayed corresponds to the order of the size of the effect. The chart includes a vertical line indicating the 95% statistical significance limit. An effect was therefore significant if the corresponding bar crossed this vertical line. According to ANOVA statistical data (Table 2) and Pareto chart of the effects for the yield of the total flavonoids (Figure 3), the quadratic terms of methanol concentration (X_1_) and the liquid/solid ratio (X_3_) had extremely significant effects on the extraction yield of TF. Those were followed by the linear term of methanol concentration (X_1_) and the quadratic terms of extraction time (X_2_). The fitness of the predicted TF model was examined by *R* square (0.9673) and by the insignificance of the lack of fit (0.3284), as shown in Table 2, suggesting a good fit with the predicted model of Equation (2). Three-dimensional response surface and contour plots for TF as a function of extraction time and methanol concentration are shown in Figure 2Aa. TF underwent a slight change with extraction time, but it greatly increased with an increase in methanol concentration, reaching a peak at a higher methanol concentration (around 80%). The linear increase in TF shown in Figure 2Ab also demonstrates that the process variables of methanol concentration and liquid/solid had significant effects on TF. With a higher liquid/solid ratio (35:1–45:1) and a higher methanol concentration (around 80%), this resulted in the highest TF yield. Figure 2Ac confirmed that liquid/solid had a significant effect on TF, whereas extraction time had relatively little effect.

Jin et al. reported an RSM optimized method for the ultrasonic-assisted extraction of total flavonoids from the fermented leaves of *A. grossedentata*, and the optimum conditions of ultrasonic-assisted extraction were as follows: the alcohol concentration was 67% (*v/v*); the ultrasonic treatment temperature was 58 °C; the ultrasonic treatment time was 28 min; and the liquid-solid ratio was 22.5:1 (v/m) [31]. It also demonstrated that the application of RSM is reliable and feasible in the UAE optimization for the extraction of flavonoids from *A. grossedentata*. However, differences of the experiment between Jin’s and ours also existed, such as the experimental materials and the extraction solvent. In Jin’s report, they used fermented *A. grossedentata* leaves as the materials and alcohol as the extraction solvent, while we used natural *A. grossedentata* leaves as the materials and menthol as the extraction solvent, respectively. Our method was more suitable for further establishing a rapid, sensitive HPLC-DAD method to simultaneously determine the contents of three bioactive compounds in *A. grossedentata*, which could have more comprehensive control and evaluate the quality for extracts from *A. grossedentata*.

#### 2.3.2. Effects of Process Variables on Dihydromyricetin Content

ANOVA statistical data (Table 2) and the Pareto chart of the effects for the dihydromyricetin yield (Figure 4) indicated that the linear terms of methanol concentration (X_1_), extraction time (X_2_), the linear and quadratic terms of liquid/solid ratio (X_3_), as well as cross product terms of extraction time (X_2_) and the liquid/solid ratio (X_3_) had significant effects on DMY. These were followed by the cross product terms of methanol concentration (X_1_) and the liquid/solid ratio (X_3_), in addition to the interaction term between methanol concentration (X_1_) and extraction time (X_2_). The coefficient of *R* square of the predicted DMY model was 0.9897, and the *p*-value for the lack of fit was 0.3656 (Table 2), suggesting a good fit with the predicted model of Equation (3). Three-dimensional surface and contour plots were generated according to Equation (3) and are shown in Figure 2B. According to Figure 2Ba, which depicts the effects of methanol concentration and extraction time on DMY, the yield of DMY obviously increased with an increase in methanol concentration. A similar linear increase in DMY yield was observed in Figure 2Bb, in which DMY increased with both methanol concentration and the liquid/solid ratio. Lastly, a significant effect of the liquid/solid ratio on DMY was observed in Figure 2Bc, as the maximum recovery of DMY was obtained at a high liquid/solid ratio (around 40:1) under the condition of an extraction time of 30 min.

#### 2.3.3. Effects of Process Variables on Myricitrin Content

According to ANOVA statistical data (Table 2) and the Pareto chart of effect for the yield of myricitrin (Figure 5), both the quadratic and linear terms of methanol concentration (X_1_) had the most significant effects on MYG (Table 2), following the linear terms of the liquid/solid ratio (X_3_), as well as the quadratic term of extraction time (X_1_) and the liquid/solid ratio (X_3_) had significant influence on the extraction yield of MYG. The coefficient of *R* square for the MYG model was 0.9472, and the *p*-value for the lack of fit was 0.8341, suggesting a good fit with the predicted model of Equation (4). The three-dimensional response surface and contour plots for MYG as a function of methanol concentration and extraction time (Figure 2Ca) indicate that MYG yield drastically increased with methanol concentration. A similar linear increase in MYG yield was observed in Figure 2Cb, in which MYG increased substantially with methanol concentration. Both the liquid/solid ratio and extraction time had positive effects on MYG as revealed in Figure 2Cc, in which the maximum recovery of MYG was also obtained at a high liquid/solid ratio (around 40:1) under the condition of an extraction time of 30 min.

#### 2.3.4. Effects of Process Variables on Myricetin Content

According to the ANOVA statistical data (Table 2) and the Pareto chart of effect for myricetin yield (Figure 6). The linear and quadratic terms of methanol concentration (X_1_) had the most significant effects on MY. In addition, the linear term of the liquid/solid ratio (X_3_) along with the interaction terms for the extraction time (X_2_) and the liquid/solid ratio (X_3_) had significant influence on the extraction yield of MY. Table 2 shows that the coefficient of *R* square of the predicted MY model was 0.9392, and the *p*-value for the lack of fit was 0.0954, suggesting a good fit with the predicted model of Equation (5). According to Figure 2Da and Figure 2Db, it is demonstrated that MY yield drastically increased with methanol concentration. However, the extraction time had no obvious effects on MYG yield. Furthermore, Figure 2Dc showed that only under the condition of a high liquid/solid ratio above (around 40:1) was there a significant effect on MY recovery, whereas a long extraction time up to 30 min had only a little effect on MY yield. 

### 2.4. Optimization of the Process Variables and Verification of the Model

**Figure 7:**
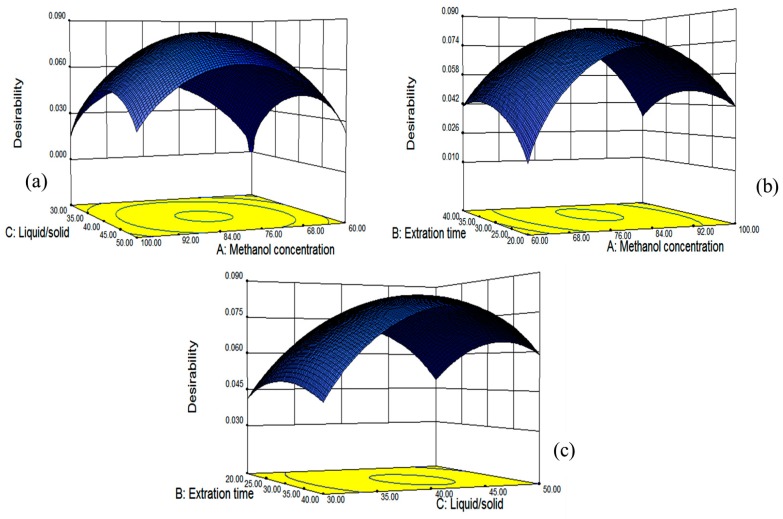
The three-dimensional plots showing the correlative effects of methanol concentration (**a**); extraction time (**b**) and the liquid/solid ratio (**c**) on the overall desirability.

The fitted model for all four responses was reliable within the region of the experiment based on the results of ANOVA, and simultaneous optimization for all responses was carried out using a Derringer’s desirability function, as shown in Figure 7. The optimum conditions given by the model were as follows: methanol concentration of 80.87%, extraction time of 31.98 min and the liquid/solid ratio of 41.64:1, which gave estimated maximal values for TF, DMY, MYG and MY. Those conditions were similar to the optimal ones from single factor tests (methanol concentration of 80%, extraction time of 30 min and liquid/solid of 40:1), which might verify that our optimization conditions of ultrasonic-assisted extraction were suitable.

Under the obtained optimum conditions, the model predicted the values for the yields of the total flavonoids and contents of three individual flavonoids along with the desirability of the conditions. Figure 7 showed the 3D plots of the response surface for the correlative effects of methanol concentration (A), extraction time (B) and liquid/solid ratio (C) on the overall desirability, which were performed by keeping one of the parameters constant at the predicted values.

In order to determine the accuracy and reliability of the predicted model, as well as to check the deviation between actual and estimated values under the proposed optimal conditions, a verification experiment was carried out under adjusted conditions (81% methanol, 32 min extraction and 21 mL methanol) on the basis of the optimal conditions. The experiment results are listed in Table 3. In addition, variations between the predicted and experimental values obtained for the contents of the total flavonoids and three compounds were depicted by the relative error (RE). No significant differences were observed between the predicted and experimental values, verifying that the fitted model for each response was valid and reliable for the simulation of UAE of flavonoids from *A. grossedentata*. In addition, the established analytical method was successfully applied to determine the contents of the total flavonoids and three individual flavonoids in 10 batches of leaf samples of *A. grossedentata* from three counties in Fujian Province, China (Supplemental Materials, Table S2). The results are shown in Table 4, which indicate variability in the quality of *A. grossedentata* leaves from different origins. This variability might be caused by different factors, such as plant gene types, habitats or harvesting times. It is necessary to further study the reasons in the future.

### 2.5. Optimization of the Solvent System and HSCCC Separation

Successful separation by HSCCC depends on the selection of a suitable two-phase solvent system, which provides an ideal range of the coefficients (*K* values) for the target compounds. A suitable *K* value range for the target compound is between 0.5 and 2.0. Small *K* values result in a loss of fraction resolution, while large *K* values tend to consume excessive solvent and a long run time [32]. The sample was extracted by the methanol–water ratios (81:19, *v*/*v*); thus, the search started with *N*-hexane–ethyl acetate–methanol–water (1:1:1:1, *v*/*v*/*v*/*v*). The result of this experiment shows that dihydromyricetin is inclined to partition well into the lower phase (mobile phase), and the peak of dihydromyricetin is close to the solvent front, which results in low purity. Then, we have to adjust the *K* value to achieve baseline separation. The *K* value could be enlarged by increasing the volume ratio of ethyl acetate versus n-hexane. When the ratio reaches four, the two-phase solvent system becomes miscible. Therefore, a series of ratios of ethyl acetate over n-hexane between one and four was performed to optimize the two-phase solvent system. It was found that a two-phase solvent system composed of *N*-hexane–ethyl acetate–methanol–water ratios (1:3:2:4, *v*/*v*/*v*/*v*) gave the optimum conditions.

**Figure 8:**
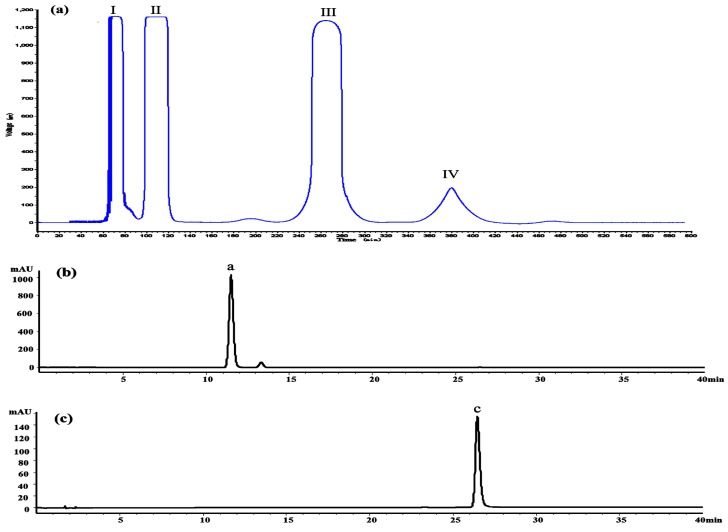
High-speed counter-current chromatography (HSCCC) chromatogram of the extracts of *Ampelopsis grossedentata* (**a**); two-phase solvent system: *N*-hexane–ethyl acetate–methanol–water (1:3:2:4, *v*/*v/v*/*v*); stationary phase: upper organic phase; mobile phase, lower aqueous phase; flow-rate: 2.0 mL/min; revolution: 800 rpm; detection wavelength: 254 nm; retention of stationary phase: 58%. HPLC chromatograms of HSCCC peak fractions, peaks of III in (**b**) dihydromyricetin, peaks of IV in (**c**) myricetin.

The crude extract was analyzed by the HPLC method at first. The result indicated that it contained several flavonoids, including dihydromyricetin (11.3 min), myricitrin (18.5 min), myricetin (26.1 min) and some other compounds, as shown in Figure 1e. Figure 8a shows the HSCCC separation chromatogram of 200 mg crude extract. Under the optimized conditions, two major components were obtained and yielded 86.46 mg of dihydromyricetin (Peak III) and 3.61 mg of myricetin (Peak IV). As shown in Figure 8b,c, the HPLC analysis of III and IV fractions revealed that two pure flavonoids could be obtained from the enriched extracts in one step elution and less than 8 h. The purities of two compounds were 95.03% and 99.21%, respectively. Their structures were confirmed by ^1^H-NMR and HPLC–ESI-Q/TOF-MS/MS data (Supplementary Materials, Table S3).

## 3. Experimental Section

### 3.1. Plant Materials

The natural leaf samples of *Ampelopsis grossedentata* were obtained from Youxi, Taining and Shanghang County of Fujian Province in China, which were air-dried under room temperature conditions. The samples’ information is listed in detail in Supplementary Materials (Table S2). All of the samples were authenticated as *Ampelopsis grossedentata* (Hand.-Mazz) W.T. Wang by Minjian Qin (Department of Resources Science of Traditional Chinese Medicines, China Pharmaceutical University, Nanjing, China). The voucher specimens were deposited in the Herbarium of Medicinal Plants of China Pharmaceutical University. Among them, Sample S3 was randomly selected as the experiments’ material for the UAE optimization and methodology studies. The dried leaves were crushed to a powder form in an electric grinder and then passed through a 40 mesh sieve. The powdered samples were stored in a freezer at 4 °C prior to analysis.

### 3.2. Chemicals and Reagents

Standard compounds of dihydromyricetin and myricetin were purchased from Chenguang Biological Technology Co., Ltd. (Baoji, China), and myricitrin was obtained from Must Biological Technology Co., Ltd. (Chengdu, China). The purity of each compound was determined to be over 98% by an HPLC-UV method. Acetonitrile and methanol of HPLC grade were purchased from Tiandi (Fairfield, CT, USA) and Hanbon (Nanjing, China), respectively. Ultra-pure water used for HPLC was produced by a Milli-Q system (Millipore, Bedford, MA, USA). All other reagents were analytical-grade reagents and obtained from Nanjing Chemical Regents Co., Ltd. (Nanjing, China).

### 3.3. Preliminary Experiments

The solvent type and extraction method directly affect the extraction efficiency and relate to the production costs, such as extraction time, solvent volume, energy costs, as well as the effect on humans and the environment. To select the best solvent for further study, three solvents are evaluated, including water (WT), ethanol (ET) and methanol (MT). Then, three different extraction methods were applied with the selected solvent (methanol), including cold-maceration extraction (CME), ultrasound-assisted extraction (UAE) and heat reflux extraction (HRE).

To investigate the effect of solvents, the extraction procedures were as follows: 0.5 g of dried material of Sample S3 was extracted with 15 mL of various solvents and subjected to UAE using an ultrasonic cleaning bath (KH5200 DB type, Kunshan ultrasonic instrument Co., Ltd., Kunshan, China) operated at a frequency of 40 kHz and at power of 200 W at 30 °C for 30 min. For the effect of the extraction methods, 0.5 g of dried material of Sample S3 were extracted with 15 mL of methanol; the extraction process included two stages; the first stage was set at room temperature (25 ± 1 °C) for 30 min, and the second stage was extracted using the experimental extraction methods; CME was carried out using a sealing conical flask at room temperature for 24 h; UAE was performed using an ultrasonic cleaner at a power of 200 W and 30 °C for 30 min; HRE was carried out using a thermostatic water bath (Buchi, Flawil, Switzerland) at 80 °C for 2 h.

After extraction, when the extracts were cooled to room temperature, the sample was centrifuged at 2000 rpm for 15 min to collect the supernatant. Then, 1 mL supernatant being diluted to 25 mL with methanol as the stock solution was used for UV detection. In addition, 2 mL supernatant were filtered through a 0.22-μm microfiltration membrane prior to HPLC analysis, which was performed by the HPLC-DAD analyses.

Based on the preliminary experiments data listed in detail in Table S1, methanol and ultrasonic-assisted extraction were selected as the solvent type and extraction method, respectively, for the enhanced extraction procedures.

### 3.4. Ultrasonic-Assisted Extraction Process

The process of UAE was performed in an ultrasonic cleaning bath (KH5200DB type, Kunshan ultrasonic instrument Co., Ltd.) operated at a frequency of 40 kHz and an ultrasonic input power of 200 W with a useable volume of 10 L. The available extraction temperature was at 30 °C. The extraction process was carried out as follows: 0.5 g powder of Sample S3 were placed in a 100-mL Erlenmeyer flask and mixed with extraction solution, after which the flask was placed in the ultrasonic bath, and extraction was carried out for the required time periods. After ultrasonic extraction, the sample was centrifuged at 2000 rpm for 15 min to collect the supernatant. Then, 1 mL supernatant being diluted to 25 mL with the extraction solvent as the stock solution was used for UV detection, and 2 mL supernatant were filtered through a 0.22-μm microfiltration membrane for HPLC analysis.

### 3.5. Determination of Total Flavonoid Content 

The total flavonoids content of *A. grossedentata* extract was determined according to the method described by Shah et al [33] with some modifications using a UV-VIS spectrophotometer (Model UV-2450, Shimadzu, Kyoto, Japan), based on the formation of a complex flavonoid-aluminum having a maximum absorbance value at 311 nm. Thus, TF was determined by using 1 mL of the stock solution mixed with 1 mL of 5% aluminum chloride (diluted to 25 mL by the extraction solvent). One millimeter of 5% aluminum chloride (added to 25 mL using extraction solvent) was used as a blank for setting at zero, and the absorption values were recorded at 311 nm. Dihydromyricetin was used as a reference for the calibration curve (Y = 0.0485X + 0.0087, *R*^2^ = 0.9998, linear range 3.68–18.40 μg/mL). The results were expressed as mg of DMY equivalent per gram (mg DMYE/g) of dried weight of the samples.

### 3.6. HPLC-DAD Analysis

High performance liquid chromatography (HPLC) analysis was conducted on an Agilent Series 1260 LC instrument (Agilent Technologies, Palo Alto, CA, USA) equipped with an on-line degasser, a quaternary pump, a diode-array detector (DAD), a thermostated column compartment and an auto-sampler. The analytes’ separation was performed on an RP-C_18_ column (Hanbon, 4.6 mm × 150 mm, 5 μm) at a flow rate of 1.0 mL/min; the sample injection volume was 10 μL; and the column temperature was continually maintained at 30 °C. The mobile phase consisted of Solvents A (0.5% aqueous formic acid, *v*/*v*) and B (acetonitrile). Gradient elution was as follows: 10%–20% B (*v*/*v*) at 0–20 min, 20%–30% B (*v*/*v*) at 20–40 min. The detecting wavelength was set at 292 nm.

The standard stock solutions were prepared by accurately dissolving weighted standards in 5 mL of pure methanol, resulting in initial concentrations of 3.072 mg/mL for dihydromyricetin (DMY), 1.518 mg/mL for myricitrin (MYG) and 0.150 mg/mL for myricetin (MY). The solutions were stored at 4 °C before being used. Standard working solutions used for the calibration were prepared by diluting the above standard solutions with methanol to the desired concentrations. The contents of the three analytes in a sample were estimated according to their respective calibration curve (DMY: Y = 2579.4X − 272.09, *R*^2^ = 0.9999, linear range 0.3072–3.072 mg/mL; MYG: Y = 966.31 + 20.264, *R*^2^ = 0.9999, linear range 0.1518–1.518 mg/mL; and MY: Y = 1146X − 16.992, *R*^2^ = 0.9998, linear range 0.0150–0.150 mg/mL).

### 3.7. Preparation of Sample and Two-Phase Solvent System for Isolation by HSCCC

Material of Sample S8 (30 g) was extracted under the optimal ultrasonic extraction conditions. After filtration, extracts were concentrated under reduced pressure with a rotary evaporator and, then, freeze-dried to produce 13.75 g of the crude extract. The sample was stored in a desiccator for the subsequent HSCCC separation.

The selected solvent system was thoroughly equilibrated in a separation funnel by repeatedly vigorously shaking for 2 min at room temperature. The two phases were separated shortly prior to use. The upper phase was used as the stationary phase, while the lower phase was used as the mobile phase. The sample solution for HSCCC separation was prepared by dissolving 200 mg crude extract in a 10-mL mixture of the lower phase and the upper phase (1:1, *v*/*v*).

### 3.8. Selection of the Two-Phase Solvent System and HSCCC Separation

The two-phase solvent system was selected according to the partition coefficient (*K* value) of each target component. *K* values were determined as follows: 2.0 mL of each phase were transferred into a test tube, and 10 mg of sample were added. The test tube was shaken for 1 min and then left for phase separation at room temperature. After two clear layers were formed (the tube was centrifuged if necessary), an aliquot (0.1 mL) of each phase was pipetted and diluted with methanol to 1.0 mL. Then, 10 μL diluted solution were injected into HPLC, and the *K* value was expressed as the ratio of the solute peak area in the upper phase (A_1_) to that in the lower phase (A_2_), *K* = (A_1_/A_2_).

In the present study, the HSCCC separation was operated on the TBE-300B high-speed counter-current chromatography (Tauto Biotechnique Company, Shanghai, China) equipped with three multilayer coil separation columns, a manual 20-mL loop sample injection valve, a TBP-5002 constant-flow pump and a TBD-2000 monitor. HSCCC separation was performed as follows: after the multi-player coiled column was entirely filled with the upper phase, then the lower phase (mobile phase) was pumped into the head of the column at a flow rate of 2.0 mL/min, while the apparatus was run at 800 rpm. The temperature of the apparatus was kept at 25 °C. After hydrodynamic equilibrium was reached, about 20.0 mL sample solution containing 200 mg of crude extract were injected through the injection valve. The column effluent was continuously monitored with a UV detector at 254 nm, and each peak fraction was collected manually according to the elution profile displayed on the recorder. Each fraction from HSCCC was analyzed by the HPLC-DAD method.

### 3.9. Statistical Analysis

All analytical experiments were performed in triplicate, and the data were expressed as mean values ± standard deviation. The regression analysis and analysis of variance (ANOVA) for the BBD test data were implemented using Design Expert software (Version 8.0.6, Stat-Ease Inc.). The statistical analyses of HPLC data were performed using ORIGIN (Version 8.0; Microcal Software Inc., Northampton, MA, USA). Pareto charts were drawn by using Minitab (Version 17.1, Minitab Inc., States Collage, PA, USA).

## 4. Conclusions

The response surface methodology was successfully implemented for the optimization of bioactive flavonoids from leaves of *A. grossedentata* using ultrasonic-assisted extraction. The methanol concentration was demonstrated to be the most significant parameter in the UAE process. Through the analysis of the response surface, it implied that methanol concentration and the liquid/solid ratio had significant effects on TF, DMY, MYG and MY yields, whereas extraction time had relatively little effects. The optimal extraction conditions were determined to be a methanol concentration of 80.87%, an extraction time of 31.98 min and a liquid/solid ratio of 41.64:1 mL/g, which gave estimated maximal values for TF, DMY, MYG and MY. The reliability of the predicted values was verified in our study, and the observed values for the responses showed no significant differences with the estimated values. Therefore, the fitted model is valid and could be used to optimize the extraction of flavonoids from *A. grossedentata*. With the optimized UAE process, we successfully determined the contents of the total flavonoids and three individual flavonoids in 10 batches of samples of *A. grossedentata*. In addition, two flavonoid aglycones, dihydromyricetin and myricetin, were separated and purified simultaneously from the extracts of *A. grossedentata* leaves under the optimum ultrasonic extraction conditions by the HSCCC method. These compounds were isolated on a sufficiently large scale with high purities in a single operation. It would be beneficial for further bioactivity research or could be used as reference substances for the quality control of the herb.

## Figures and Tables

**Table 1:** Experimental values of the response variables for the Box–Behnken design (BBD).

Runs	Independent Variables ^a^			Dependent Variables ^b^ (Mean ± SD) *n* = 3			
	X_1_	X_2_	X_3_	TF	DMY	MYG	MY
1	80 (0)	20 (−1)	30:1 (−1)	280.72 ± 8.80	130.62 ± 0.78	30.30 ± 0.64	2.66 ± 0.12
2	60 (−1)	20 (−1)	40:1 (0)	273.88 ± 4.25	142.63 ± 1.49	30.00 ± 0.90	1.84 ± 0.05
3	100 (1)	20 (−1)	40:1 (0)	289.84 ± 0.15	143.06 ± 0.29	26.73 ± 1.28	2.50 ± 0.07
4	80 (0)	30 (0)	40:1 (0)	306.15 ± 9.02	147.98 ± 3.34	32.23 ± 0.45	2.61 ± 0.05
5	80 (0)	30 (0)	40:1 (0)	299.15 ± 6.24	145.39 ± 0.45	35.23 ± 0.18	2.71 ± 0.05
6	80 (0)	40 (1)	50:1 (1)	287.66 ± 4.07	139.57 ± 0.60	31.72 ± 0.04	3.04 ± 0.02
7	100 (1)	30 (0)	30:1 (−1)	284.53 ± 5.73	132.24 ± 1.36	24.17 ± 0.68	2.21 ± 0.05
8	80 (0)	30 (0)	40:1 (0)	306.94 ± 5.74	146.94 ± 0.21	35.70 ± 0.15	2.76 ± 0.01
9	60 (−1)	40 (1)	40:1 (0)	279.81 ± 2.79	143.63 ± 0.79	30.63 ± 0.28	2.02 ± 0.03
10	100 (1)	30 (0)	50:1 (1)	273.31 ± 8.88	136.01 ± 1.12	27.37 ± 0.04	2.42 ± 0.01
11	80 (0)	30 (0)	40:1 (0)	304.18 ± 6.35	145.24 ± 0.12	35.00 ± 0.13	2.79 ± 0.01
12	100 (1)	40 (1)	40:1 (0)	283.48 ± 11.63	151.36 ± 0.31	26.08 ± 1.41	2.34 ± 0.09
13	60 (−1)	30 (0)	30:1 (−1)	271.32 ± 1.99	122.67 ± 0.42	30.47 ± 0.22	2.12 ± 0.05
14	80 (0)	20 (−1)	50:1 (1)	287.29 ± 2.43	130.19 ± 0.28	34.81 ± 1.10	2.56 ± 0.09
15	80 (0)	40 (1)	30:1 (−1)	290.68 ± 6.48	129.19 ± 0.34	30.78 ± 0.25	2.46 ± 0.02
16	80 (0)	30 (0)	40:1 (0)	305.13 ± 3.57	146.93 ± 0.60	35.83 ± 0.67	2.79 ± 0.02
17	60 (−1)	30 (0)	50:1 (1)	271.28 ± 9.78	134.96 ± 0.21	31.47 ± 0.35	2.24 ± 0.02

^a^ Independent variables: X_1_, methanol concentration (%); X_2_, extraction time (min); X_3_, liquid/solid ratio (mL/g). ^b^ Dependent variables: TF, total flavonoids (mg/g); DMY, dihydromyricetin (mg/g); MYG, myricitrin content (mg/g); and MY, myricetin content (mg/g).

**Table 2:** ANOVA statistics of quadratic models for the extraction yields of the total flavonoids (TF), dihydromyricetin (DMY), myricitrin (MYG) and myricetin (MY) from *A. grossedentata* leaves.

Term	Source	Mean Square	*F*-Value	*p*-Value	Significant
TF ^a^	Model	269.16	23.00	0.0002	significant
	X_1_	151.99	12.99	0.0087	**
	X_1_^2^	1219.35	104.20	<0.0001	**
	X_2_^2^	129.23	11.04	0.0127	*
	X_3_^2^	624.90	53.40	0.0002	**
	Lack of fit	14.77	1.57	0.3284	not significant
DMY ^b^	Model	117.70	74.64	<0.0001	significant
	X_1_	44.09	27.95	0.0011	**
	X_2_	32.70	23.59	0.0018	**
	X_3_	84.57	53.62	0.0002	**
	X_1_X_2_	13.32	8.45	0.0228	*
	X_1_X_3_	18.15	11.51	0.0116	*
	X_2_X_3_	29.21	18.52	0.0035	**
	X_3_^2^	813.72	515.98	<0.0001	**
	Lack of fit	1.88	1.40	0.3656	not significant
MYG ^c^	Model	21.07	13.96	0.0011	significant
	X_1_	41.50	27.49	0.0012	**
	X_3_	11.64	7.71	0.0274	*
	X_1_^2^	104.64	69.32	<0.0001	**
	X_2_^2^	8.89	5.89	0.0457	*
	X_3_^2^	8.76	5.81	0.0468	*
	Lack of fit	0.62	0.29	0.8341	not significant
MY ^d^	Model	0.17	12.01	0.0017	significant
	X_1_	0.20	14.07	0.0072	**
	X_3_	0.082	5.91	0.0454	*
	X_2_X_3_	0.12	8.33	0.0234	*
	X_1_^2^	1.03	74.26	<0.0001	**
	Lack of fit	0.025	4.33	0.0954	not significant

^a^ The *R* square obtained in fit statistics for the response model of TF was 0.9673; ^b^ the *R* square obtained in fit statistics for the response model of DMY was 0.9897; ^c^ the *R* square obtained in fit statistics for the response model of MYG was 0.9472; ^d^ the *R* square obtained in fit statistics for the response model of MY was 0.9392. * Significant (*p* < 0.05). ** Extremely significant (*p* < 0.01).

**Table 3:** Predicted and experimental values of the responses obtained under the optimal extraction conditions. RE, relative error.

	Methanol Concentration (%)	Extraction Time (min)	Liquid/Solid Ratio (mL/g)	Contents ^a^ (mg/g)				
				TF	DMY	MYG	MY	Desirability
Predicted	80.87	31.98	41.64:1	303.89	147.26	34.70	2.76	0.082
Experimental	81	32	42:1	312.69	151.61	33.25	2.70	
RE (%)				2.81	2.87	4.36	2.22	

^a^ The contents of total flavonoids and three compounds are expressed as mg/g of plant on a dry weight basis. Experimental values are given as the mean (*n* = 3).

**Table 4:** Contents of the total flavonoids, dihydromyricetin, myricitrin and myricetin in *Ampelopsis grossedentata* leaves from different origins.

Contents ^a^ (mg/g)				
Samples	TF	DMY	MMG	MY
S1	322.97 ± 1.66	152.82 ± 0.60	22.77 ± 1.11	4.16 ± 0.03
S2	399.70 ± 1.81	183.44 ± 4.22	28.34 ± 0.92	2.06 ± 0.10
S3	314.85 ± 8.30	141.54 ± 1.91	32.32 ± 1.35	2.72 ± 0.13
S4	525.61 ± 6.75	192.67 ± 1.06	51.77 ± 0.19	2.74 ± 0.08
S5	471.94 ± 10.75	186.22 ± 1.64	46.89 ± 0.42	2.18 ± 0.02
S6	438.92 ± 9.18	195.18 ± 0.89	23.56 ± 0.41	2.47 ± 0.07
S7	477.49 ± 4.19	174.74 ± 0.98	64.80 ± 0.29	1.87 ± 0.02
S8	535.41 ± 2.54	205.00 ± 0.42	40.98 ± 0.34	3.21 ± 0.08
S9	302.79 ± 9.16	99.99 ± 0.50	39.18 ± 0.27	4.13 ± 0.03
S10	334.38 ± 9.43	115.95 ± 0.24	45.52 ± 0.94	4.60 ± 0.03

^a^ The contents of total flavonoids and three compounds are expressed as mg/g of plant on a dry weight basis. Values are given as the mean ± SD (*n = 3*). The samples information shown in Table S2.

## Supplementary Materials

Supplementary materials can be accessed at: http://www.mdpi.com/1420-3049/21/8/1096/s1.
