# Supplementary material for: Optimization of the Ultrasonic-Assisted Extraction of Bioactive Flavonoids from Ampelopsis grossedentata and Subsequent Separation and Purification of Two Flavonoid Aglycones by High-Speed Counter-Current Chromatography
Source: Molecules. 2016 Aug 20;21(8):1096. doi: 10.3390/molecules21081096 (PMC6273339; doi:10.3390/molecules21081096)
Supplement: Supplementary file 1 [file molecules-21-01096-s001.pdf]

Supplementary Materials: Optimization of the Ultrasonic-Assisted Extraction of Bioactive Flavonoids from *Ampelopsis grossedentata* and Subsequent Separation and Purification of Two Flavonoid Aglycones by High-Speed Counter-Current Chromatography

Table S1. The extraction yields of total flavonoid and three compounds obtained using different solvent and extraction methods.

Solvent/Extraction Method	Extraction Yield (mg/g) [c]			
	**Total Flavonoid**	**Dihydromyricetin**	**Myricitrin**	**Myricetin**
Water [a]	152.15 ± 7.65	49.77 ± 10.07	5.05 ± 0.47	0.40 ± 0.01
Ethanol [a]	212.26 ± 19.38	130.79 ± 5.79	7.22 ± 1.39	1.24 ± 0.19
Methanol [a]	291.10 ± 2.33	132.20 ± 2.45	26.22 ± 1.83	2.47 ± 0.04
CME [b]	257.38 ± 4.34	124.77 ± 0.33	18.00 ± 0.41	3.20 ± 0.04
HRE [b]	325.40 ± 4.79	121.05 ± 0.31	35.35 ± 0.50	1.29 ± 0.06
UAE [b]	300.82 ± 5.96	133.93 ± 0.26	26.27 ± 0.70	2.50 ± 0.04

[a] The extraction procedures were as follows: 0.5 g of dried sample was extracted with 15 mL of various solvents applying UAE. [b] Extraction methods: cold-maceration extraction (CME), heat reflux extraction (HRE), and ultrasound-assisted extraction (UAE). [c] The extraction yields of total flavonoids and three compounds are expressed as mg/g of plant dry weight basis. Values are given as the mean ± sd ($n = 3$).

Table S2. The information of samples of *Ampelopsis grossedentata* collected from different origins.

Code	Name	Source	Longitude and Latitude, Altitude	Collection Date
S-1	*A. grossedentata.*	Youxi, Fujian	N 26″10′51.21 E 118″10′28.44 H 399m	15 April 2015
S-2	*A. grossedentata.*	Youxi, Fujian	N 26″07′50.31 E 118″06′54.66 H 133m	16 April 2015
S-3	*A. grossedentata.*	Youxi, Fujian	N 26″09′42.93 E 118″10′52.97 H 388m	17 April 2015
S-4	*A. grossedentata.*	Taining, Fujian	N 26″46′54.79 E 117″01′24.29 H 349m	20 April 2015
S-5	*A. grossedentata.*	Taining, Fujian	N 27″02′21.71 E 117″05′48.87 H 631m	21 April 2015
S-6	*A. grossedentata.*	Taining, Fujian	N 26″48′32.20 E 117″08′51.72 H 465m	23 April 2015
S-7	*A. grossedentata.*	Shanghang, Fujian	N 24″50′01.95 E 117″34′40.08 H 180m	24 April 2015
S-8	*A. grossedentata.*	Shanghang, Fujian	N 24″58′30.10 E 116″42′34.51 H 465m	25 April 2015
S-9	*A. grossedentata.*	Youxi, Fujian	N 26″19′16.06 E 118″11′03.48 H 212m	25 August 2015
S-10	*A. grossedentata.*	Youxi, Fujian	N 26″13′13.95 E 118″01′42.51 H 410m	26 August 2015

^{1}H-NMR and HPLC–ESI-Q/TOF-MS/MS Analysis of Isolated Dihydromyricetin and Myricetin by HSCCC

Dihydromyricetin and myricetin isolated and purified by the high-speed counter-current chromatography (HSCCC) were confirmed by ^{1}H-NMR and HPLC–ESI-Q/TOF-MS/MS analysis, the data shown in Table S3.

The ^{1}H-NMR experiment was performed on a Bruker ACF-500M NMR spectrometer (Bruker, Rheinstetten, Germany) using DMSO as solvent. HPLC–ESI-Q/TOF-MS/MS analysis was performed by an Agilent series 1290 HPLC instrument (Agilent, Waldbronn, Germany) coupled with an Agilent 6530 Q-TOF mass spectrometer (Agilent Technologies, Santa Clara, CA, USA) equipped with an ESI ion source as interface. The mobile phase consisted of (A) 0.5% aqueous formic acid and (B) acetonitrile. The gradient program and detection wavelength were the same with HPLC-DAD analyses. The mass spectra were acquired across the range of *m/z* 100–1700 in negative mode. The operating parameters of mass spectrometer were as follows: drying gas (N_2) flow rate, 8.0 L/min; drying gas temperature, 320 °C; nebulizer, 40 psig; capillary voltage, 3000 V; fragment voltage 120 V; skimmer voltage, 60 V and Oct RFV, 750 V. The collision energy was set at 30 V. The MS data was controlled by MassHunter software B.04.00 ChemStation (Agilent Technologies).

Table S3. MS and ^{1}H-NMR and HPLC–ESI-Q/TOF-MS/MS data of component III, IV isolated by HSCCC method.

Component	MS (*m/z*)			δ Values of ^{1}H-NMR (500 MHz) in DMSO-d_6
	Molecular Formula	Quasi-Molecular (error, ppm)	Molecular Mass	At 500 MHz
Peak (III) (dihydromyricetin)	$C_{15}H_{12}O_8$	319.0459 (2.31) [M − H]$^-$	320.0532	11.90 (1H, s, C5-OH)<br>10.79 (1H, s, OH)<br>8.87 (2H, s, C3′, 5′-OH)<br>8.18 (1H, s, OH)<br>5.74 (1H, d, *J* = 6.0 Hz, C3-OH)<br>5.88 (1H, d, *J* = 2.0 Hz, H-6)<br>5.93 (1H, d, *J* = 2.4 Hz, H-8)<br>4.93 (1H, d, *J* = 10.7 Hz, H-2)<br>4.43 (1H, dd, *J* = 11. 0, 5.6 Hz, H-3)<br>6.42 (2H, s, H-2', 6')
Peak (IV) (myricetin)	$C_{15}H_{10}O_8$	317.0303 (−0.97) [M − H]$^-$	318.0376	12.51 (1H, s, C5-OH)<br>7.23 (2H, s, H-2′, 6′)<br>6.21 (1H, d, *J* = 2.1 Hz, H-6)<br>6.40 (1H, d, *J* = 2.1 Hz, H-8)
